# Acute, Subacute, and Genotoxicity Assessments of a Proprietary Blend of *Garcinia mangostana* Fruit Rind and *Cinnamomum tamala* Leaf Extracts (CinDura®)

**DOI:** 10.1155/2020/1435891

**Published:** 2020-07-30

**Authors:** Sundararaju Dodda, Venkata Krishnaraju Alluri, Trimurtulu Golakoti, Krishanu Sengupta

**Affiliations:** Laila Nutraceuticals R&D Center, Vijayawada, Andhra Pradesh, India

## Abstract

The present communication describes a battery of toxicity studies that include an acute oral toxicity, a subacute twenty-eight-day repeated oral dose toxicity, and genotoxicity studies on a herbal formulation CinDura® (GMCT). This proprietary herbal composition contains the extracts of the *Garcinia mangostana* fruit rind (GM) and the *Cinnamomum tamala* leaf (CT). The toxicological evaluations were performed following the Organization for Economic Cooperation and Development (OECD) guidelines. The acute oral toxicity study in Wistar rats suggests that the median lethal dose of CinDura® is at least 2000 mg/kg body weight. Acute dermal and eye irritation tests in New Zealand white rabbits indicate that the test item is nonirritant to the skin and eyes. A twenty-eight-day repeated dose oral toxicity study was conducted in male and female Wistar rats using daily doses of 250, 500, and 1000 mg/kg body weight, followed by a fourteen-day reversal period for two satellite groups. The CinDura®-supplemented animals did not show any sign of toxicity on their body weights, organ weights, and on the hematobiochemical parameters. The gross pathology and histopathological examinations indicated no treatment-related changes in the experimental animals. Overall, the no-observed-adverse-effect level (NOAEL) of the herbal blend is 1000 mg/kg body weight, the highest tested dose. Also, the results of the bacterial reverse mutation test and the erythrocyte micronucleus assay in mouse bone marrow suggest that CinDura® (GMCT) is neither mutagenic nor clastogenic.

## 1. Introduction

CinDura® (GMCT) is a proprietary botanical composition; it contains aqueous-ethanol extracts of the *Garcinia mangostana* fruit rind (GM) and *Cinnamomum tamala* leaf (CT). A preclinical study demonstrated that CinDura®-supplemented Swiss albino mice had improved grip strength and swimming performance in a forced swim test. Further, a double-blind, placebo-controlled clinical study established that GMCT enhanced muscle strength and endurance among young males when supplemented in combination with a resistance training program [1].

*Garcinia mangostana*, popularly known as mangosteen, grows in the Asian region such as in Malaysia, Myanmar, Thailand, Philippines, Sri Lanka, and India. The fruit contains soft and juicy edible pulp [2]. The pericarp of this fruit or its fruit rind is used as a traditional medicine in treating various ailments such as trauma, skin infection, abdominal pain, dysentery, and wounds [3]. A major xanthone, *α*-mangostin, is rich in the *G. mangostana* fruit rind extract [4]. *α*-Mangostin is a potent bioactive phytochemical responsible for anti-inflammatory, analgesic, antioxidant, and antilipogenic activities [3, 4]. *Cinnamomum tamala* is commonly known as tejpatta, Malabar leaf, or Indian bay leaf in India. *C. tamala* leaves are widely used as a flavoring agent and spice in a variety of culinary preparations and as a natural food preservative in the Asian countries [5]. In Ayurveda, these leaves have a high medicinal value. Traditionally, *C tamala* leaves are used to treat diabetes, hyperlipidemia, inflammation, hepatotoxicity, diarrhea, etc. [6, 7]. *C. tamala* leaves extracts, or its essential oil, were shown to have potential anti-inflammatory, antioxidant, antimicrobial, antidiabetic, and hepatoprotective effects in *in vitro* and *in vivo* models [8].

Despite extensive use of various preparations of *G. mangostana* fruit rind or *C. tamala* leaves as medicine or food, the toxicological evaluations of GMCT are vital to establish its safety for human use. Earlier, Jujun et al. reported that repeated oral supplementation of an ethanol extract of *G. mangostana* fruit rind for twenty-eight days did not show systemic toxicity in Sprague Dawley rats, which concluded its safety for human consumption [9]. However, to the best of our knowledge, no report is available so far on preclinical toxicological evaluations, including genetic toxicity studies on *C tamala* leaves. Therefore, it is crucial to conduct a systemic and genetic toxicological assessment to ensure the safety of the herbal blend for human consumption although the individual ingredients are believed to be safe.

Here, we present acute oral toxicity and a repeated dose 28-day oral toxicity studies in Wistar rats to establish the systemic safety of GMCT. Further, a bacterial reverse mutation assay and a micronucleus assay in mouse bone marrow erythrocytes present the genetic safety of the blend. All experiments followed the testing guidelines of the Organization for Economic Cooperation and Development (OECD).

## 2. Materials and Methods

### 2.1. Test Item

The test item GMCT (CinDura® or LI80020F4) is a proprietary herbal composition manufactured in a Good Manufacturing Practice- (GMP-) certificated facility of Laila Nutraceuticals, Andhra Pradesh, India. It is composed of seven parts of an herbal blend containing aqueous ethanol extracts of the *Garcinia mangostana* (GM) fruit rind and *Cinnamomum tamala* (CT) leaf at 1 : 2 ratio and three parts of the excipients, microcrystalline cellulose and Syloid. The final blend CinDura® is a light greenish-brown to dark brown powder with a characteristic odor and taste and is standardized to contain at least 3.5% *α*-mangostin and 0.1% rutin, as described earlier [1]. The manufacturing process included extraction and processing of the individual plant raw materials under appropriate process controls and comprised typical process steps including pulverizing, extraction, concentration, and drying, followed by blending of extracts along with the excipients and sieving. The final product was tested for residual solvents, heavy metals, and microbial growth as part of the quality control check.

The plant raw materials, *G. mangostana* fruit rind and *C. tamala* leaves, were purchased from Indonesia and Nainital, Uttarakhand, India, respectively. Following the taxonomic identification of the plant raw materials, Laila Nutraceuticals R&D Center, Vijayawada, India, preserved their voucher specimens. The methods of extract preparations and the phytochemical standardization of GMCT are described earlier [1].

### 2.2. Chemicals, Reagents, and Bacterial Strains

Sodium carboxymethyl cellulose (CMC-Na), dimethyl sulfoxide (DMSO), nicotine-adenine dinucleotide phosphate (NADP), glucose-6-phosphate (G6P), sodium azide, 2-nitrofluorene, 9-aminoacridine, 4-nitroquinoline-1-oxide, 2-aminoanthracene, fetal bovine serum (FBS), absolute methanol, cyclophosphamide monohydrate (CPA), sodium thiopentone barbiturate, isoflurane, magnesium chloride, potassium chloride, and sodium phosphate salts were obtained from Sigma-Aldrich (St. Louis, MO). *Salmonella typhimurium* TA98, TA100, TA1535, and TA1537 strains were sourced from the National Collection of Type Cultures (London, UK). *Escherichia coli* WP2 *uvr*A/pKM101 was obtained from the National Collection of Industrial, Food, and Marine Bacteria (Scotland, UK). Lyophilized rat liver S9 fraction and Bacto Agar were purchased from Celsis International (Cambridge, UK) and Becton Dickinson, Sparks, MD, respectively. The clinical chemistry and hematology reagents were obtained from Instrumentation Laboratory India Pvt. Ltd. (New Delhi, India) and Siemens (Munich, Germany), respectively.

### 2.3. Experimental Animals

Pathogen-free adult 7-8 week-old male and female Wistar rats were purchased from Vivo Bio Tech Ltd., Hyderabad, India. Male and female Swiss albino mice (6–8 weeks old) were obtained from Palamur Biosciences Pvt. Ltd., Hyderabad, India. New Zealand white male rabbits (9–10 weeks of age, weighing 2.0–2.8 kg) were procured from Mahaveera Enterprises, Hyderabad, India. The animals were acclimatized to the housing conditions (22 ± 3°C with 40–70% relative humidity, and in a 12 h light-dark cycle) for one week before the start of the experiments. The animals received a standard rodent chow and reverse osmosis-filtered water *ad libitum*. All procedures related to animal handling and investigations followed the Committee for the Purpose of Control and Supervision of Experiments on Animals (CPCSEA) guidelines for animal care. The Institutional Animal Ethics Committee (IAEC), Laila Nutraceuticals, Andhra Pradesh, India, approved the study protocols.

### 2.4. Acute Oral Toxicity Study

An acute oral toxicity test was conducted in overnight-fasted female Wistar rats (180–200 g body weight), following the OECD Test 425 guidelines [10]. The code number of the ethical document of this study is LN/IAEC/TOX/LN140405. A limit test was performed by sequential use of five female Wistar rats in an interval of 48 hrs. GMCT was dissolved in distilled water, and a single dose of 2000 mg/kg was administered through oral gavage. Following the dose administration, the animals were monitored for any clinical signs of toxicity every hour for the first four hours and then every day for the next 14 days. All animals' body weight, food, and water consumptions were recorded daily. Following the CO_2_ euthanasia at day 15, the vital organs and tissues were examined for any gross pathological changes.

### 2.5. Acute Dermal Toxicity Study

An acute dermal toxicity test (ethics approval document number: LN/IAEC/TOX/LN140104) was conducted in five male (weighing 239–264 g) and five female (weighing 219–235 g) Wistar rats using a single dermal application of GMCT (2000 mg/kg BW), following the OECD Test Guideline 402 [11]. In brief, GMCT was moistened with distilled water and evenly distributed on a gauze patch. The gauze patch was then applied to the intact skin (4 × 4 cm) and held securely using a nonirritating adhesive tape for 24 h. After the patch was removed, the residual test item was wiped off the skin using water-soaked cotton. The animals were observed for signs of toxicity after 30 min of application, at every hour up to 6 hr and then daily for consecutive 14 days. The animals were examined for any gross pathological changes.

### 2.6. Acute Dermal Irritation Study

The acute dermal irritation test (ethical approval document number: LN/IAEC/TOX/LN141203) was performed on healthy young adult male New Zealand white rabbits (2.0-2.25 kg), following the OECD Test Guideline 404 [12]. Five hundred milligram of moistened GMCT was applied on the shaved skin at the dorsal trunk area of the rabbits with the help of a gauge pad (6 × 6 cm). The gauge pad was securely held on the skin using a nonirritating adhesive tape for 4 hr. After removal of the gauge pad, the signs of skin reactions were observed for up to 72 hr. First, the test procedure was performed on one rabbit and then repeated on two more rabbits. After the observation period, the animals were sacrificed; their vital organs were examined for any gross pathological changes.

### 2.7. Acute Eye Irritation Study

The acute eye irritation potential of GMCT was tested (ethical document code number: LN/IAEC/TOX/LN141204) in healthy young male rabbits, following the OECD Test Guideline 405 [13]. Before the test item application, the eyes were examined; 100 mg of the test item was applied in the conjunctival sacs of their left eyes. Following the dosing, the eyelids were held together for a few seconds to prevent the loss of the test item. After 1 hr, the treated eye was rinsed with sterile saline. The untreated right eye served as the control. The signs of eye irritation in the conjunctiva, iris, and cornea of both eyes were scored at 1, 24, 48, and 72 hr after the test item application using an ophthalmoscope [14]. First, the test procedure was performed on one rabbit and then repeated on two more rabbits. After the observation period, the animals were sacrificed; their vital organs were examined for any gross pathological changes.

### 2.8. Twenty-Eight-Day Repeated Dose Oral Toxicity Study

A twenty-eight-day repeated dose oral toxicity study (ethics approval document number: LN/IAEC/TOX/LN141206) was conducted in Wistar rats, following the OECD guidelines 407 [15]. Rats of both sexes (*n* = 10; five males and five females) were randomly allocated into six groups—four primary groups and two reversal groups. GMCT prepared in 0.5% CMC-Na was administered daily for twenty-eight days in single oral doses of 250 (low dose, G2), 500 (mid dose, G3), or 1000 mg/kg BW (high dose, G4). The vehicle control group (G1) received 0.5% CMC-Na with no test item (0) through oral gavage daily for 28 days. On day 29, the animals in the primary groups G1, G2, G3, and G4 were euthanized by CO_2_ inhalation. The two reversal groups, G1R and G4R, received oral gavages of 0.5% CMC-Na containing no test item (0) and 1000 mg/kg BW/day GMCT, respectively, for twenty-eight days and were sustained in their in-life phase with regular rodent chow for an additional fourteen days, without the vehicle or GMCT supplementation. The dose verification and homogeneity of the test product were analyzed using HPLC on days 1 and 25 of the study. All dose formulations were prepared fresh every day and administered at an equal dosing volume of 10 mL/kg BW. The body weight was recorded weekly and at necropsy; the clinical signs, morbidity, or mortality were recorded every day during the study duration. Body weight and feed consumption were measured weekly and at necropsy.

At necropsy, the animals were fasted overnight, and blood samples were collected through the retro-orbital puncture (under isoflurane anesthesia) for hematology and clinical chemistry analysis. The hematobiochemical parameters were measured using a hematology analyzer ADVIA 2120 (Siemens Healthcare Private Limited, Munich, Germany) and a clinical chemistry analyzer (ILab Aries, Bergamo, Italy). The hematology parameters included red blood cells (RBCs), white blood cells (WBCs), neutrophils (Neu), lymphocytes (Lym), eosinophils (Eos), monocytes (Mono), basophils (Baso), platelets (Plt), reticulocytes (Rec), hemoglobin concentration (Hb), hematocrit (Hct), mean corpuscular volume (MCV), mean corpuscular hemoglobin (MCH), and mean corpuscular hemoglobin concentration (MCHC). The serum biochemical parameters included glucose (Glu), blood urea nitrogen (BUN), urea (Ur), total cholesterol (T.Chol), triglycerides (Trig), total bilirubin (T.Bil), aspartate aminotransferase (AST), alanine aminotransferase (ALT), alkaline phosphatase (ALP), total protein (TP), albumin (Alb), calcium (Ca), phosphorus (Phos), sodium (Na), potassium (K), and chloride (Cl).

After CO_2_ euthanasia, the animals' vital organs were collected for macroscopic and microscopic pathological examinations. The organs harvested were the liver, kidneys, heart, lungs, brain, spleen, adrenal glands, thymus, testes, epididymis, seminal vesicles, ovaries, and uterus. The organs were weighed on an electronic balance with 0.01 g accuracy (Mettler Toledo, Columbus, OH). For histopathology examinations, the organs were fixed in 10% neutral buffered formalin for 48 hr. The paraffin-embedded organs were cut into 5 *μ*m thick sections using a rotary microtome. The tissue sections were processed in graded alcohol and stained with hematoxylin and eosin. The stained tissue sections were examined under a light microscope Axioscope (Carl Zeiss, Munich, Germany).

### 2.9. Bacterial Reverse Mutation Test

The bacterial reverse mutation test was conducted following the OECD Test Guideline 471 [16]. The mutagenic effect of GMCT was assessed in *S. typhimurium* TA98, TA100, TA1535, TA1537 strains and *Escherichia coli* WP2 *uvr*A (pKM101) in the presence or absence of metabolic activation (S9 fraction) [17, 18]. In brief, a hundred microliters of fresh broth of each bacterial strain was mixed with increasing concentrations of the test product. The bacterial suspensions were mixed with an overlay agar and plated over a minimal glucose agar plate. The plates were incubated at 37°C for 48–72 hr. The final concentrations of GMCT were 100, 266, 707, 1880, and 5000 *μ*g per plate. The vehicle control culture plates received sterile normal saline mixed with the bacterial culture broth. Each dose of GMCT was tested in three replicate plates. The number of revertant colonies was counted manually in the test plates. In parallel, the cultures grown in the presence or absence of the strain-specific mutagens, 2-aminoanthracene or 2-nitrofluorence or sodium azide or 9-aminoacridine or 4-nitroquinoline-1-oxide, were positive controls. A test concentration was considered as mutagenic only when the number of revertant colonies was at least double the vehicle control (spontaneous yield).

### 2.10. *In Vivo* Micronucleus Assay

Mammalian erythrocyte micronucleus assay was conducted in healthy Swiss albino mice (6–8 weeks old), following the OECD Test Guideline 474 [19]. Male and female mice were divided into five groups (*n* = 10; five males and five females). The animals received GMCT through oral gavage at doses of 500 (G2), 1000 (G3), or 2000 mg/kg BW (G4) mixed in 0.5% CMC-Na. The vehicle control group (G1) animals received 0.5% w/v CMC-Na through oral gavage. The test item and the vehicle were administered two times at an interval of 24 hrs. The positive control animals (G5) received cyclophosphamide monohydrate (40 mg/kg) through intraperitoneal (*i.p.*) injection only once on the second day, 24 hr before euthanasia. All animals were examined for clinical signs. Following CO_2_ euthanasia, the bone marrow samples were aspirated from both femurs and smeared on randomly coded glass microscope slides. The Giemsa-stained bone marrow samples were examined under 40x objective of a light microscope (ECLIPSE E200, Nikon Corporation, Tokyo, Japan). Polychromatic erythrocytes (PCE) and normochromic erythrocytes (NCE) were counted in each bone marrow sample. A total of 4000 polychromatic erythrocytes (PCEs) were scored in each sample. The frequency of micronucleated polychromatic erythrocytes (MNPCEs) was expressed as a percentage. Besides, the number of PCE was counted in 1000 total number of erythrocytes (TE = PCE + NCE), and a ratio between PCE and TE represented the frequency of PCE.

### 2.11. Statistical Analyses

The data were expressed as mean ± SD. Comparison analyses among different groups were performed by analysis of variance using the one-way analysis of variance (ANOVA) test with Dunnett's post hoc test. Student's *t*-test was used to analyze the data from the reversal groups for comparison. All comparisons were evaluated at the 95% level of confidence, and *P* values less than 0.05 were considered statistically significant.

## 3. Results

### 3.1. A Single-Dose Oral Administration of GMCT Did Not Cause Abnormality or Death in Wistar Rats

No morbidity or mortality occurred following a single-dose oral administration of 2000 mg/kg BW GMCT in the female Wistar rats. The rats were generally active and did not show any visible signs of toxicity. They did not show abnormal changes in their body weights, feed, and water consumption during the postdose 14-day observation period. A gross pathological examination did not show any abnormalities. Together, these observations indicate that the median oral lethal dose (LD_50_) of GMCT is at least 2000 mg/kg BW in female Wistar rats.

### 3.2. GMCT Did Not Show Signs of Toxicity or Irritation in the Skin or Eyes

The GMCT-treated male and female Wistar rats did not show any signs of dermal toxicity, adverse pharmacological effects, or any behavioral abnormality. These results suggest that that the dermal LD_50_ of the test item is at least 2,000 mg/kg BW in Wistar rats of either sex.

GMCT did not show any skin reactions in the New Zealand rabbits in the acute dermal irritation test. The observations suggest that the acute skin irritation index is 0 and conclude that GMCT is nonirritant to the rabbit skin [20].

In the eye irritation test, application of GMCT did not show signs of severe irritation in the experimental animals. The iris was normal throughout the observation period. There were no signs of ulceration or opacity in the cornea. Conjunctival redness and mild swelling or chemosis were observed in the test eye of the experimental rabbits; however, these signs were reversible, and the eyes became normal within 24 hr of application. Based on the Harmonized Integrated Classification System, GMCT is nonirritant to the eyes [20].

### 3.3. GMCT Supplementation Was Nontoxic and Did Not Yield Pharmacologic Effects in the 28-Day Repeated Dose Oral Toxicity Study

Oral administration of GMCT for 28 days did not attribute any sign of toxicity and mortality in male and female Wistar rats. The rats in the primary groups, including the reversal groups, were generally healthy and active. All animals in the primary and reversal groups survived through the end of the study. Tables 1 and 2 present the total and weekly feed consumption by the male and female rats in the primary and reversal groups. Among the primary groups, the male high-dose group (G4 male) consumed significantly less feed in the first week of the study, compared with the control (G1 male) rats. The G4R males had significantly less feed during the first three weeks and the final week of the study. The G4R female rats also consumed substantially less feed in the first week of the study, compared with the respective control rats (G1R female). However, the total feed consumption by the treated rats was not significantly different compared with the matched controls irrespective of the sex of animals or treatments. The changes in body weights of male and female rats following the test item supplementation are presented in Tables 3 and 4, respectively. GMCT-supplemented male and female rats in the primary groups including the reversal groups did not show treatment-related changes in their overall body weight when compared with the vehicle control animals. All groups of GMCT-supplemented male and female rats showed a natural pattern of body weight gain as observed in the vehicle control rats during the study (Tables 3 and 4). At necropsy, the vital organs of the experimental rats were weighed. The absolute and the relative organ weights (expressed as a percentage of their body weights) of the male and female rats are shown in Tables 5 and 6, respectively. The absolute and relative organ weights of the GMCT-supplemented male and female rats were not statistically different when compared to the sex-matched controls, except a significant increase in total but not in the relative weight of the spleen in G4R females.

No treatment-related significant changes were observed in the hematology parameters of GMCT-supplemented male and female rats (Tables 7 and 8), with an exception that the leucocyte count significantly increased in the G4 female rats, compared with the control rats (Table 8).

The serum biochemistry parameters of the male and female experimental rats are presented in Tables 9 and 10, respectively. The mid dose (G3) male rats showed a significantly reduced level of total protein, compared with the control (G1 male). The other biochemical parameters did not show treatment-related changes (Table 9). Overall, in the females, the majority of the biochemical parameters were not significantly different from the controls, with the exceptions of variations in AST, ALT, total cholesterol, and glucose (Table 10). The G2 and G3 female rats showed an increased AST level; and the G4 female rats showed an increased ALT level, compared with the control (G1 female) rats. Serum glucose and total cholesterol were significantly increased in the G4R female rats when compared with the G1R rats. No such treatment-related changes were observed in the male rats of primary or reversal groups of the study. However, the observed levels of these biochemical parameters are within the normal ranges. The male and female rats did not show treatment-related changes in the kidney function parameters (creatinine and BUN). GMCT supplementation did not affect the major serum electrolytes, calcium, magnesium, sodium, and potassium.

### 3.4. GMCT Supplementation Did Not Cause Significant Macroscopic or Microscopic Changes in the Vital Organs of the Male and Female Rats

**Figure 1:**
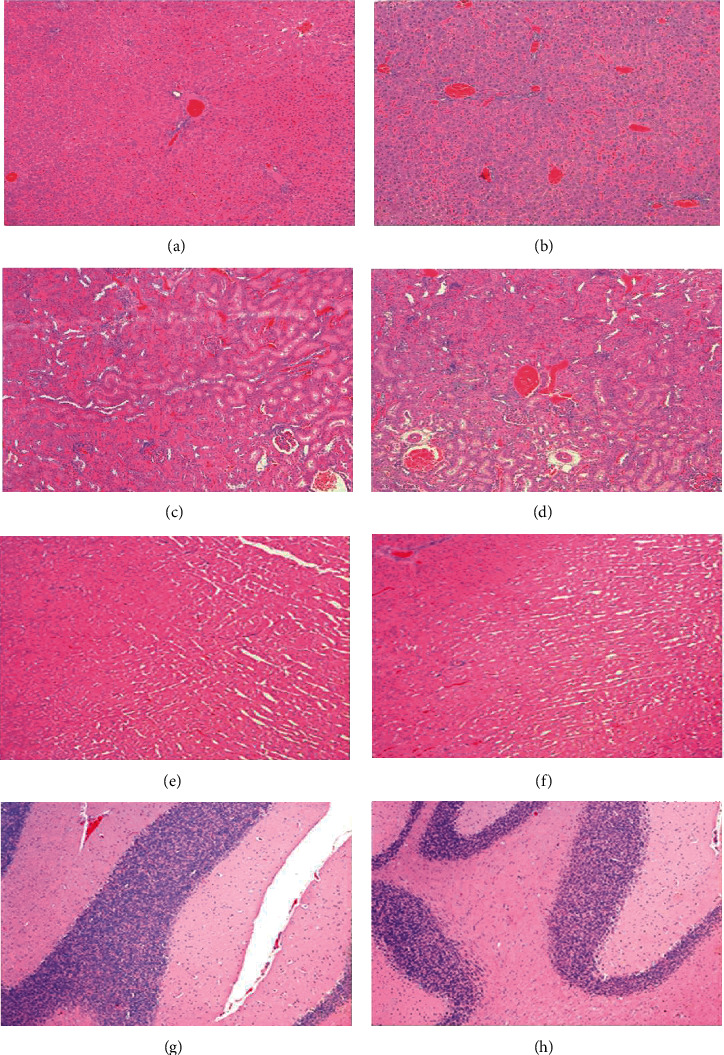
Photomicrographs showing representative hematoxylin-eosin-stained (100x) sections of the liver, kidney, heart, and brain of the control and 1000 mg/kg GMCT-supplemented rats in the 28-day toxicity study. The left panels (a), (c), (e), and (g) present the liver, kidney, heart, and brain of the control rats, respectively. The right panels (b), (d), (f), and (h) present the liver, kidney, heart, and brain of the GMCT-1000-supplemented rats, respectively. There are no treatment-related microscopic changes in the major tissues of GMCT-supplemented rats compared to the control rats.

Daily administration of 1000 mg/kg BW GMCT did not cause substantial changes in the vital organs of the experimental male and female rats. The gross morphology of the vital organs of the high-dose male and female rats was unaltered following the treatment and reversal period. Tables 11 and 12 summarize the histological observations on the vital organs of the male and female animals, respectively. The GMCT-supplemented rats showed no significant histological changes compared with the control rats. With an exception, one rat in both sexes showed mild traces of mineralization in the kidneys following the test item supplementation. Overall, the treatment-related effects on the vital organs were similar to those of the control rats. The microscopic examinations did not show significant changes in the hematoxylin-eosin-stained sections of the vital organs of the high-dose-supplemented rats compared to the control rats (Figure 1).

### 3.5. GMCT Is Neither Mutagenic nor Clastogenic *In Vitro* and *In Vivo*

The Ames bacterial reverse mutation assay showed that the increasing concentrations of GMCT did not alter the number of revertant colonies of *S. typhimurium* and *E. coli* strains in the presence or absence of the S9 metabolic activation (Table 13). The strain-specific mutagens, served as the positive controls, significantly increased the number of revertant colonies in the culture plates as indicated (Table 13).

In the micronucleus assay, GMCT supplementation did not increase the ratio of the polychromatic erythrocyte (PCE) and the total erythrocyte (TE) in the bone marrow samples of either male or female mice, in comparison with the vehicle control animals. Besides, GMCT administration also did not increase the frequency of the micronucleated PCE (MNPCE) in male or female mice. The positive control groups or the cyclophosphamide-treated mice showed a significant increase in the incidence of MNPCE in their bone marrow samples (Table 14).

## 4. Discussion

The current study evaluated the safety and toxicological profile of GMCT (CinDura®), a proprietary composition of *Garcinia mangostana* fruit rind and *Cinnamomum tamala* leaf extracts. Individually, these two plant raw materials have precious medicinal value in complementary and alternative medicine since centuries [3, 5, 6]. Besides, these plant materials are considered safe based on the long history of human consumption either as a medicine or food ingredient. In the majority, the usage history of the traditional medicinal herbs determines their safety. Following the OECD guidelines, we assessed the toxicity of the herbal blend in an acute oral, acute dermal toxicity/irritation, and a subacute twenty-eight-day repeated dose oral toxicity studies in Wistar rats. A primary eye irritation study in rabbits evaluated the irritant or corrosive effects of GMCT. Besides, the bacterial reverse mutation assay and a micronucleus assay in mouse bone marrow erythrocytes evaluated the genetic toxicity of this herbal composition.

GMCT (CinDura®) is a proprietary combination of aqueous ethanol extracts of the *G. mangostana* fruit rind and *C. tamala* leaf at 1 : 2 ratio. This herbal blend is standardized to contain at least 3.5% *α*-mangostin and 0.1% rutin [1]. A double-blind, placebo-controlled clinical trial demonstrated that 800 mg/day of GMCT supplementation significantly improved muscle strength, muscle growth, and endurance performance in the young male participants in conjunction with a resistance training schedule of six weeks [1]. Besides, a series of *in vitro* experiments demonstrated that GMCT activated endothelial nitric oxide synthase in human endothelial cells and improved mitochondrial biogenesis in rat skeletal myoblasts. Moreover, this composition also activated mTOR signaling in the rat myoblasts *in vitro* (to be published elsewhere).

In the present study, we first determined that the oral LD_50_ of GMCT was 2000 mg/kg BW of Wistar rats, the highest tested dose. This herbal composition did not show any acute toxicity or mortality or any abnormalities in hematobiochemical parameters; the gross pathology of the vital organs was also unaltered. According to the toxicological classification criteria of the OECD, the observations suggest that the test item is a nontoxic composition and falls in the “no label” category [21]. Further, GMCT did not show any signs of dermal irritation or toxicity. Also, based on the “Harmonized Integrated Classification System for Human Health and Environmental Hazards of Chemical Substances and Mixtures,” the herbal blend is nonirritant to the eyes.

In the twenty-eight-day subacute toxicity study followed by the fourteen-day reversal period, the highest dose of 1000 mg/kg/day of GMCT-supplemented rats did not show visible signs of toxicity. No animal in the primary or the reversal groups died during the study. The animals did not show treatment-related changes in body weight, cumulative feed consumptions, gross anatomy, organ weights, or histopathology. The toxic chemicals reduce body weight and cause associated changes in the absolute and relative organ weights [22]. In the present subacute study, all animals in the treatment groups gradually gained body weights throughout the study. Although some evaluation points showed that the G2, G4, and G4R rats of both sexes consumed significantly less feed in comparison with the respective controls, those were neither consistent nor dose dependent. The amount of feed consumed by these groups did not correlate with their body weight data; also, the total feed consumption in these groups was not significantly different compared with the respective controls. Together, these observations suggest that the oral administration of GMCT did not affect the natural growth and metabolism of the experimental rats.

Hematology and serum biochemical parameters are essential for understanding the overall physiological status of the body, investigating a disease condition, diagnosis, and liver toxicity [23, 24]. In the present study, the GMCT-supplemented rats (in the primary and reversal groups) did not show significant changes in the hematobiochemical parameters, compared with the respective control rats with exceptions in some parameters, most importantly AST and ALT in female rats in the primary groups of the study. However, these hematology and clinical chemistry values are within the normal physiological ranges related to the sex and age of Wistar rats [25]. These changes are neither related to the dose nor the duration of the treatment. Elevated levels beyond the typical ranges of the liver transaminases indicate the abnormal liver function or the liver injury caused by toxic chemical exposure [26]. *C. tamala* leaves extracts are potent antioxidants and are hepatoprotective against chemical-induced toxicity in rodents [8, 27]. Recently, Fu et al. reported that alpha-mangostin from the *G. mangostana* fruit rind extract activated antioxidant defense and parallelly induced anti-inflammatory response to protect lipopolysaccharide/*d*-galactosamine-induced liver toxicity in mice [28]. Kidneys eliminate urea and creatinine from the body through urine. Elevated levels of these metabolites in circulation indicate lack of clearance from the body due to impaired kidney function [29]. In the present study, oral administration of GMCT did not affect the serum urea and creatinine levels in the male and female rats, indicating no treatment-related adverse effect on the kidneys of the experimental rats. Overall, the observations on the hematobiochemical parameters suggest that oral administration of the maximum tested dose of the herbal blend (1000 mg/kg/day) did not yield pathologic changes in the vital organs of the treated rats.

Further, the gross and microscopic examinations of the vital organs revealed no treatment-related changes in the rats. These observations also support that oral administration of this herbal composition did not cause systemic toxicity in the male and female rats. Taken together, the results of the twenty-eight-day repeated oral toxicity study establish that the “no-observed-adverse-effect level (NOAEL)” of GMCT (CinDura®) in the male and female rats is 1000 mg/kg body weight per day, the highest tested dose. This dose is equivalent to 162.07 mg/kg or a daily dose of 9604 mg for a human subject of 60 kg body weight, at least twelve-fold higher than the dose used in the clinical study [1].

Besides, a bacterial reverse mutation assay and a micronucleus assay in mouse bone marrow erythrocytes evaluated the genotoxic potential of GMCT. The toxicological data of genotoxicity assays are essential components of the safety evaluation of a drug candidate [30]. In the present study, the negative results from the Ames test and the *in vivo* micronucleus assay suggested that the test item did not induce mutagenesis and also did not promote clastogenesis or DNA damage. Hence, GMCT consumption is not of genotoxicity concern. Together, based on the results of the battery of toxicity studies, we conclude that the oral use of GMCT (LI80020F4 or CinDura®) is expected to be safe for humans.

## Figures and Tables

**Table 1:** Effect of oral administration of GMCT on feed consumption by male Wistar rats.

Feed consumption (g/week/animal)						
Days	Primary groups Dose (mg/kg/day)				Reversal groups Dose (mg/kg/day)	
	0 (G1)	250 (G2)	500 (G3)	1000 (G4)	0 (G1R)	1000 (G4R)
1–7	137.51 ± 6.90	137.87 ± 0.26	134.96 ± 3.80	128.21 ± 4.24^*∗*^	141.31 ± 1.98	132.87 ± 7.47^#^
8–14	133.36 ± 3.48	138.87 ± 8.91	141.85 ± 10.06	132.77 ± 13.93	140.93 ± 1.50	132.00 ± 7.50^#^
15–21	139.57 ± 10.18	148.31 ± 0.49	140.68 ± 8.66	131.80 ± 12.31	139.51 ± 1.41	128.81 ± 4.90^#^
22–28	123.55 ± 4.85	135.28 ± 4.61^*∗*^	123.09 ± 4.13	119.27 ± 14.62	143.83 ± 1.80	143.09 ± 4.66
29–35	—	—	—	—	144.51 ± 1.48	139.84 ± 8.30
36–42	—	—	—	—	123.37 ± 1.15	117.14 ± 5.67^#^

Total feed consumption from day 1 (g, mean ± SD)						
	533.99 ± 25.41	563.30 ± 14.27	540.58 ± 26.65	512.05 ± 45.10	833.46 ± 9.32	793.75 ± 38.51

Data are present as mean ± SD; *n* = 5. ^*∗*^ and ^#^Significance *p* < 0.05 vs. G1 and G1R, respectively.

**Table 2:** Effect of oral administration of GMCT on feed consumption by female Wistar rats.

Feed consumption (g/week/animal)						
Days	Primary groups Dose (mg/kg/day)				Reversal groups Dose (mg/kg/day)	
	0 (G1)	250 (G2)	500 (G3)	1000 (G4)	0 (G1R)	1000 (G4R)
1–7	94.80 ± 0.51	93.68 ± 5.75	94.86 ± 0.40	96.74 ± 1.53	95.74 ± 1.48	92.84 ± 2.02^#^
8–14	100.56 ± 6.62	96.00 ± 3.04	98.97 ± 1.13	101.34 ± 0.58	100.08 ± 0.41	94.27 ± 6.39
15–21	99.50 ± 3.26	105.24 ± 4.57	99.59 ± 2.53	99.89 ± 4.64	97.00 ± 2.0	96.03 ± 4.40
22–28	88.55 ± 0.65	90.29 ± 0.77^*∗*^	87.32 ± 0.88	90.62 ± 1.59^*∗*^	99.78 ± 5.04	90.12 ± 10.59
29–35	—	—	—	—	97.53 ± 5.87	101.47 ± 2.58
36–42	—	—	—	—	80.15 ± 3.37	83.16 ± 2.98

Total feed consumption from day 1 (g, mean ± SD)						
	383.41 ± 11.04	385.21 ± 14.13	380.74 ± 4.94	388.59 ± 8.34	570.28 ± 18.17	557.89 ± 28.96

Data are present as mean ± SD; *n* = 5. ^*∗*^ and ^#^Significance *p* < 0.05 vs. G1 and G1R, respectively.

**Table 3:** Effect of oral administration of GMCT on body weight of male Wistar rats.

Body weight (g, mean ± SD)						
Days	Primary groups Dose (mg/kg/day)				Reversal groups Dose (mg/kg/day)	
	0 (G1)	250 (G2)	500 (G3)	1000 (G4)	0 (G1R)	1000 (G4R)
1	260.61 ± 14.33	260.59 ± 6.65	263.02 ± 20.70	258.95 ± 7.25	262.28 ± 10.72	261.01 ± 14.04
8	289.73 ± 14.02	291.27 ± 14.42	291.52 ± 26.48	287.06 ± 11.55	292.11 ± 15.22	288.21 ± 15.15
15	314.99 ± 16.23	315.35 ± 20.81	318.79 ± 31.42	313.08 ± 15.89	315.95 ± 17.37	311.09 ± 15.45
22	334.91 ± 18.80	336.06 ± 22.66	337.89 ± 35.66	328.23 ± 17.97	339.49 ± 20.78	323.87 ± 18.55
29	348.71 ± 20.82	354.05 ± 21.53	352.89 ± 36.29	341.04 ± 24.04	356.20 ± 27.92	344.86 ± 13.79
36	—	—	—	—	374.39 ± 30.85	362.61 ± 17.25
42	—	—	—	—	385.89 ± 31.55	370.08 ± 17.74

Data are present as mean ± SD; *n* = 5; no significance.

**Table 4:** Effect of oral administration of GMCT on body weight of female Wistar rats.

Body weight (g, mean ± SD)						
Days	Primary groups Dose (mg/kg/day)				Reversal groups Dose (mg/kg/day)	
	0 (G1)	250 (G2)	500 (G3)	1000 (G4)	0 (G1R)	1000 (G4R)
1	191.02 ± 10.29	187.96 ± 7.89	190.62 ± 6.25	192.01 ± 7.56	188.94 ± 5.79	187.78 ± 4.65
8	198.40 ± 10.69	199.47 ± 11.34	201.79 ± 8.18	205.58 ± 11.15	201.98 ± 4.75	202.15 ± 5.13
15	214.09 ± 12.37	208.21 ± 15.88	212.11 ± 8.73	222.43 ± 12.23	210.84 ± 8.05	212.95 ± 9.43
22	221.66 ± 13.27	215.44 ± 15.26	221.15 ± 8.44	230.02 ± 12.34	218.33 ± 8.21	219.98 ± 7.81
29	226.23 ± 13.13	221.62 ± 17.36	226.48 ± 9.29	233.97 ± 11.99	224.26 ± 8.30	228.13 ± 4.21
36	—	—	—	—	229.14 ± 8.66	235.74 ± 6.46
43	—	—	—	—	233.02 ± 8.80	239.06 ± 6.49

Data are present as mean ± SD; *n* = 5; no significance.

**Table 5:** Effect of oral administration of GMCT on absolute and relative organ weights of male Wistar rats.

Organs	Primary groups Dose (mg/kg/day)				Reversal groups Dose (mg/kg/day)	
	0 (G1)	250 (G2)	500 (G3)	1000 (G4)	0 (G1R)	1000 (G4R)
Liver						
Absolute weight (g)	10.98 ± 1.26	11.69 ± 1.04	11.05 ± 1.59	10.27 ± 1.57	10.74 ± 1.20	10.25 ± 1.30
% body weight	3.29 ± 0.25	3.48 ± 0.34	3.28 ± 0.25	3.14 ± 0.28	2.90 ± 0.10	2.87 ± 0.23
Kidney						
Absolute weight (g)	2.33 ± 0.24	2.35 ± 0.14	2.38 ± 0.37	2.24 ± 0.24	2.44 ± 0.26	2.31 ± 0.20
% body weight	0.70 ± 0.04	0.70 ± 0.03	0.71 ± 0.05	0.69 ± 0.05	0.66 ± 0.05	0.65 ± 0.03
Adrenals						
Absolute weight (g)	0.06 ± 0.01	0.05 ± 0.01	0.06 ± 0.01	0.06 ± 0.01	0.06 ± 0.01	0.06 ± 0.00
% body weight	0.02 ± 0.00	0.02 ± 0.00	0.02 ± 0.00	0.02 ± 0.00	0.02 ± 0.00	0.02 ± 0.00
Heart						
Absolute weight (g)	1.09 ± 0.13	1.11 ± 0.15	1.09 ± 0.15	0.98 ± 0.06	1.02 ± 0.07	1.02 ± 0.08
% body weight	0.33 ± 0.04	0.33 ± 0.04	0.32 ± 0.03	0.3 ± 0.02	0.28 ± 0.01	0.29 ± 0.02
Brain						
Absolute weight (g)	2.01 ± 0.14	1.95 ± 0.07	1.98 ± 0.11	2.01 ± 0.09	2.07 ± 0.05	2.03 ± 0.15
% body weight	0.60 ± 0.02	0.58 ± 0.04	0.59 ± 0.04	0.62 ± 0.04	0.56 ± 0.04	0.57 ± 0.03
Spleen						
Absolute weight (g)	0.68 ± 0.08	0.99 ± 0.60	0.61 ± 0.09	0.68 ± 0.07	0.70 ± 0.08	0.67 ± 0.12
% body weight	0.20 ± 0.01	0.30 ± 0.19	0.18 ± 0.02	0.21 ± 0.02	0.19 ± 0.01	0.19 ± 0.03
Thymus						
Absolute weight (g)	0.44 ± 0.07	0.39 ± 0.07	0.38 ± 0.11	0.41 ± 0.10	0.35 ± 0.03	0.31 ± 0.04
% body weight	0.13 ± 0.02	0.12 ± 0.02	0.11 ± 0.03	0.12 ± 0.02	0.09 ± 0.01	0.09 ± 0.01
Testes						
Absolute weight (g)	3.59 ± 0.35	3.39 ± 0.30	3.44 ± 0.41	3.24 ± 0.46	3.61 ± 0.23	3.71 ± 0.19
% body weight	1.08 ± 0.08	1.01 ± 0.07	1.02 ± 0.10	0.99 ± 0.11	0.98 ± 0.13	1.04 ± 0.04
Epididymides						
Absolute weight (g)	1.11 ± 0.12	1.07 ± 0.09	1.03 ± 0.15	0.96 ± 0.17	1.21 ± 0.14	1.26 ± 0.08
% body weight	0.33 ± 0.03	0.32 ± 0.04	0.31 ± 0.02	0.29 ± 0.04	0.33 ± 0.05	0.35 ± 0.02
Seminal vesicles and prostate						
Absolute weight (g)	2.07 ± 0.32	2.32 ± 0.43	2.15 ± 0.41	2.10 ± 0.11	2.17 ± 0.18	2.23 ± 0.26
% body weight	0.62 ± 0.10	0.69 ± 0.15	0.64 ± 0.13	0.65 ± 0.08	0.59 ± 0.07	0.63 ± 0.09

*n* = 5; data are present as mean ± SD; no significance.

**Table 6:** Effect of oral administration of GMCT on absolute and relative organ weights of female Wistar rats.

Organs	Primary groups Dose (mg/kg/day)				Reversal groups Dose (mg/kg/day)	
	0 (G1)	250 (G2)	500 (G3)	1000 (G4)	0 (G1R)	1000 (G4R)
Liver						
Absolute weight (g)	6.57 ± 0.68	6.57 ± 1.12	6.79 ± 0.31	7.29 ± 0.89	6.24 ± 0.60	6.56 ± 0.22
% body weight	3.07 ± 0.20	3.16 ± 0.37	3.17 ± 0.06	3.28 ± 0.23	2.88 ± 0.37	2.76 ± 0.34
Kidneys						
Absolute weight (g)	1.59 ± 0.15	1.49 ± 0.12	1.61 ± 0.13	1.63 ± 0.11	1.46 ± 0.12	1.60 ± 0.13
% body weight	0.74 ± 0.05	0.72 ± 0.06	0.75 ± 0.04	0.73 ± 0.03	0.67 ± 0.07	0.67 ± 0.11
Adrenal glands						
Absolute weight (g)	0.07 ± 0.02	0.07 ± 0.01	0.06 ± 0.01	0.08 ± 0.01	0.07 ± 0.01	0.07 ± 0.01
% body weight	0.03 ± 0.01	0.03 ± 0.00	0.03 ± 0.00	0.03 ± 0.00	0.03 ± 0.00	0.03 ± 0.00
Heart						
Absolute weight (g)	0.73 ± 0.05	0.67 ± 0.07	0.71 ± 0.03	0.71 ± 0.09	0.74 ± 0.02	0.73 ± 0.04
% body weight	0.34 ± 0.02	0.33 ± 0.02	0.33 ± 0.00	0.32 ± 0.03	0.34 ± 0.02	0.31 ± 0.04
Brain						
Absolute weight (g)	1.84 ± 0.13	1.83 ± 0.11	1.81 ± 0.10	1.91 ± 0.05	1.89 ± 0.07	1.91 ± 0.10
% body weight	0.86 ± 0.03	0.89 ± 0.03	0.85 ± 0.02	0.86 ± 0.06	0.87 ± 0.06	0.80 ± 0.08
Spleen						
Absolute weight (g)	0.48 ± 0.08	0.46 ± 0.05	0.48 ± 0.06	0.56 ± 0.08	0.45 ± 0.03	0.56 ± 0.08^*#*^
% body weight	0.22 ± 0.03	0.22 ± 0.01	0.22 ± 0.02	0.25 ± 0.03	0.21 ± 0.02	0.23 ± 0.05
Thymus						
Absolute weight (g)	0.41 ± 0.05	0.33 ± 0.07	0.36 ± 0.05	0.37 ± 0.03	0.30 ± 0.08	0.37 ± 0.06
% body weight	0.19 ± 0.03	0.16 ± 0.03	0.17 ± 0.02	0.17 ± 0.02	0.14 ± 0.04	0.16 ± 0.04
Uterus with CrV						
Absolute weight (g)	0.45 ± 0.05	0.52 ± 0.11	0.41 ± 0.02	0.43 ± 0.12	0.59 ± 0.25	0.50 ± 0.21
% body weight	0.21 ± 0.02	0.25 ± 0.05	0.19 ± 0.01	0.19 ± 0.05	0.27 ± 0.11	0.21 ± 0.10
Ovaries						
Absolute weight (g)	0.12 ± 0.02	0.10 ± 0.04	0.09 ± 0.01	0.10 ± 0.02	0.09 ± 0.01	0.09 ± 0.01
% body weight	0.05 ± 0.01	0.05 ± 0.01	0.04 ± 0.00	0.04 ± 0.01	0.04 ± 0.01	0.04 ± 0.01

^*#*^ *p* < 0.05 vs. G1R *n* = 5; data are present as mean ± SD.

**Table 7:** Effect of oral administration of GMCT on hematology parameters in male Wistar rats.

Parameters	Primary groups Dose (mg/kg/day)				Reversal groups Dose (mg/kg/day)	
	0 (G1)	250 (G2)	500 (G3)	1000 (G4)	0 (G1R)	1000 (G4R)
RBC (10^6^/*μ*L)	9.26 ± 0.22	9.01 ± 0.45	9.20 ± 0.58	9.46 ± 0.33	9.21 ± 0.24	9.46 ± 0.65
Hb (g/dL)	16.54 ± 0.23	16.00 ± 0.44	16.32 ± 0.87	16.62 ± 0.50	15.96 ± 0.50	16.28 ± 0.50
Hct (%)	50.68 ± 1.18	48.82 ± 1.35	49.60 ± 2.34	50.82 ± 0.73	49.38 ± 2.14	50.22 ± 2.60
MCV (fL)	54.70 ± 0.72	54.26 ± 1.51	53.96 ± 2.6	53.78 ± 1.41	53.56 ± 1.45	53.14 ± 1.43
MCH (pg)	17.88 ± 0.26	17.76 ± 0.53	17.78 ± 0.86	17.58 ± 0.36	17.32 ± 0.26	17.26 ± 0.73
MCHC (g/dL)	32.66 ± 0.32	32.70 ± 0.44	32.94 ± 0.65	32.7 ± 0.55	32.32 ± 0.37	32.5 ± 0.78
Plt (10^3^/*μ*L)	859.8 ± 39.8	859.60 ± 117.10	851.80 ± 50.60	817.4 ± 153.77	801.2 ± 134.29	817.6 ± 174.19
WBC (10^3^/*μ*L)	9.12 ± 0.86	8.97 ± 3.68	8.08 ± 1.71	7.2 ± 1.99	8.27 ± 0.78	7.27 ± 0.99
Neu (%)^$^	12.16 ± 0.76	23.06 ± 10.26^*∗*^	19.70 ± 5.01	15.2 ± 2.22	18.36 ± 3.61	16.76 ± 2.83
Lym (%)^$^	83.44 ± 1.63	71.44 ± 11.42^*∗*^	75.00 ± 5.56	80.66 ± 2.43	76.78 ± 3.71	78.06 ± 2.35
Mono (%)	2.10 ± 0.48	2.96 ± 0.81	2.72 ± 0.94	2.14 ± 0.59	2.64 ± 0.40	2.6 ± 0.91
Eos (%)	1.20 ± 0.46	1.22 ± 0.56	1.10 ± 0.44	1.04 ± 0.28	1.42 ± 0.77	1.56 ± 0.54
Baso (%)	0.42 ± 0.04	0.44 ± 0.23	0.36 ± 0.11	0.38 ± 0.18	0.38 ± 0.13	0.42 ± 0.11
LUC (%)	0.68 ± 0.11	0.90 ± 0.50	1.08 ± 0.48	0.56 ± 0.23	0.42 ± 0.13	0.58 ± 0.22
Rec (%)	2.40 ± 0.37	2.44 ± 0.36	2.34 ± 0.24	2.40 ± 0.40	2.32 ± 0.23	2.48 ± 0.23
CT (sec)	130.40 ± 18.7	146.6 ± 18.60	149.40 ± 20.31	134.00 ± 17.90	127.00 ± 10.42	142.6 ± 26.15

Data are present as mean ± SD; *n* = 5. ^*∗*^Significance *p* < 0.05 vs. G1. ^$^The mean ± SD of the historical normal values of neutrophils (%) is 14.90 ± 3.69 (min. 8.20%; max. 30.20%) and lymphocytes (%) is 80.01 ± 4.31 (min. 63.0%; max. 87.80%).

**Table 8:** Effect of oral administration of GMCT on hematology parameters in female Wistar rats.

Parameters	Primary groups Dose (mg/kg/day)				Reversal groups Dose (mg/kg/day)	
	0 (G1)	250 (G2)	500 (G3)	1000 (G4)	0 (G1R)	1000 (G4R)
RBC (10^6^/*μ*L)	8.70 ± 0.35	8.81 ± 0.47	8.56 ± 0.34	8.94 ± 0.29	8.67 ± 0.55	8.75 ± 0.24
Hb (g/dL)	15.64 ± 0.97	15.78 ± 0.65	15.62 ± 0.74	15.86 ± 0.53	15.62 ± 0.91	15.66 ± 0.35
Hct (%)	47.62 ± 2.86	47.96 ± 1.25	46.82 ± 2.33	48.66 ± 1.29	47.94 ± 2.84	48.70 ± 1.03
MCV (fL)	54.7 ± 1.20	54.52 ± 2.09	54.64 ± 1.52	54.46 ± 0.99	55.30 ± 1.02	55.66 ± 0.55
MCH (pg)	17.96 ± 0.42	17.94 ± 0.48	18.22 ± 0.40	17.76 ± 0.54	18.02 ± 0.24	17.90 ± 0.51
MCHC (g/dL)	32.82 ± 0.29	32.90 ± 0.94	33.36 ± 1.03	32.62 ± 0.43	32.58 ± 0.28	32.12 ± 0.90
Plt (10^3^/*μ*L)	929.00 ± 46.69	806.20 ± 56.4	810.60 ± 314.78	1000.40 ± 136.02	834.80 ± 112.17	915.80 ± 102.40
WBC (10^3^/*μ*L)	5.32 ± 0.78	4.55 ± 1.15	5.24 ± 1.20	5.61 ± 1.20	4.85 ± 0.84	5.68 ± 1.68
Neu (%)	11.30 ± 1.77	20.82 ± 7.44	18.52 ± 12.38	12.92 ± 3.39	13.66 ± 4.42	12.38 ± 2.82
Lym (%)	84.10 ± 1.66	74.26 ± 8.23	75.96 ± 12.82	82.32 ± 3.21	81.24 ± 4.76	82.68 ± 2.88
Mono (%)	1.84 ± 0.31	2.48 ± 0.96	2.48 ± 0.50	2.20 ± 0.42	2.26 ± 0.65	2.26 ± 0.30
Eos (%)	1.98 ± 0.31	1.60 ± 0.44	2.18 ± 0.87	1.26 ± 0.23	1.74 ± 0.78	1.52 ± 0.51
Baso (%)	0.26 ± 0.09	0.24 ± 0.05	0.28 ± 0.13	0.30 ± 0.10	0.34 ± 0.09	0.28 ± 0.16
LUC (%)^$^	0.52 ± 0.16	0.60 ± 0.10	0.64 ± 0.11	1.00 ± 0.33^*∗*^	0.76 ± 0.35	0.92 ± 0.43
Rec (%)	2.32 ± 0.11	2.04 ± 0.17	2.16 ± 0.26	2.46 ± 0.38	2.50 ± 0.40	2.28 ± 0.23
CT (sec)	136.60 ± 25.55	144.60 ± 21.07	146.00 ± 13.80	142.60 ± 32.55	118.00 ± 14.58	122.60 ± 9.29

Data are present as mean ± SD; *n* = 5. ^*∗*^*p* < 0.05 vs. G1. ^$^The mean ± SD of the historical normal values (%) of large unstained cells (LUC) is 1.20 ± 0.6 (min. 0.3%; max. 4.0%).

**Table 9:** Effect of oral administration of GMCT on biochemical parameters in male Wistar rats.

Parameters	Primary groups Dose (mg/kg/day)				Reversal groups Dose (mg/kg/day)	
	0 (G1)	250 (G2)	500 (G3)	1000 (G4)	0 (G1R)	1000 (G4R)
Glu (mg/dL)	150.40 ± 12.54	120.60 ± 14.57	144.40 ± 42.00	126.80 ± 12.28	132.00 ± 10.93	123.20 ± 10.10
BUN (mg/dL)	17.40 ± 2.41	18.40 ± 2.07	16.60 ± 4.16	20.20 ± 3.19	15.00 ± 1.00	15.40 ± 1.82
Ur (mg/dL)	37.60 ± 5.86	39.00 ± 4.12	35.60 ± 8.73	43.60 ± 7.09	32.00 ± 1.58	33.00 ± 3.87
Crea (mg/dL)	0.50 ± 0.03	0.48 ± 0.02	0.45 ± 0.05	0.49 ± 0.02	0.46 ± 0.05	0.47 ± 0.03
T.Chol (mg/dL)	53.40 ± 6.23	48.00 ± 3.67	52.40 ± 4.56	46.40 ± 7.27	53.20 ± 6.42	51.60 ± 13.07
Trig (mg/dL)	210 ± 82.5	173.4 ± 79.17	173.8 ± 88.3.5	161.00 ± 19.21	162.00 ± 44.23	154.40 ± 23.96
T.Bil (mg/dL)	0.04 ± 0.02	0.06 ± 0.04	0.04 ± 0.02	0.04 ± 0.02	0.12 ± 0.02	0.09 ± 0.03
AST (U/L)	88.20 ± 1.80	106.20 ± 18.86	88.20 ± 8.29	88.60 ± 8.80	99.60 ± 12.76	101.20 ± 13.65
ALT (U/L)	45.00 ± 2.92	53.60 ± 15.92	46.80 ± 14.24	61.60 ± 8.76	58.60 ± 6.54	55.80 ± 9.98
ALP (U/L)	109.00 ± 26.90	118.00 ± 16.87	103.00 ± 21.64	97.20 ± 12.70	85.40 ± 7.16	101.60 ± 16.23
TP (g/dL)^$^	6.88 ± 0.26	6.76 ± 0.15	6.46 ± 0.19^*∗*^	6.58 ± 0.26	6.86 ± 0.38	6.76 ± 0.09
Alb (g/dL)	4.06 ± 0.18	4.00 ± 0.12	3.94 ± 0.05	3.98 ± 0.25	3.96 ± 0.13	3.94 ± 0.05
Ca (mg/dL)	10.58 ± 0.15	10.38 ± 0.43	10.14 ± 0.15	10.14 ± 0.52	10.56 ± 0.28	10.22 ± 0.19
Phos (mg/dL)	6.90 ± 1.33	6.48 ± 0.81	6.32 ± 0.58	6.60 ± 0.67	6.50 ± 0.89	6.10 ± 0.64
Na (mmoL/L)	141.86 ± 0.40	141.38 ± 0.98	141.66 ± 0.81	142.44 ± 0.80	142.32 ± 0.87	142.14 ± 0.36
K (mmoL/L)	3.78 ± 0.18	3.82 ± 0.23	3.83 ± 0.17	3.74 ± 0.11	3.64 ± 0.12	3.86 ± 0.19
Cl (mmoL/L)	104.48 ± 0.77	104.10 ± 1.51	104.4 ± 1.11	104.28 ± 1.30	105.04 ± 1.62	104.96 ± 0.90

Data are present as mean ± SD; *n* = 5. ^*∗*^Significance *p* < 0.05 vs. G1. ^$^The mean ± SD of the historical normal values total protein (TP) is 6.23 ± 0.33 g/dL (min. 5.50 g/dL; max. 6.90 g/dL).

**Table 10:** Effect of oral administration of GMCT on biochemical parameters in female Wistar rats.

Parameters	Primary groups Dose (mg/kg/day)				Reversal groups Dose (mg/kg/day)	
	0 (G1)	250 (G2)	500 (G3)	1000 (G4)	0 (G1R)	1000 (G4R)
Glu (mg/dL)	110.60 ± 10.48	107.80 ± 7.46	99.80 ± 8.76	109.00 ± 10.95	102.40 ± 5.03	118.0 ± 13.32#
BUN (mg/dL)	17.80 ± 0.45	19.60 ± 4.51	21.40 ± 3.91	19.40 ± 2.70	18.80 ± 2.05	20.80 ± 2.17
Ur (mg/dL)	38.80 ± 1.10	42.00 ± 9.14	45.60 ± 8.32	41.60 ± 6.19	40.00 ± 3.94	44.40 ± 4.39
Crea (mg/dL)	0.54 ± 0.04	0.55 ± 0.04	0.52 ± 0.06	0.55 ± 0.05	0.54 ± 0.04	0.55 ± 0.08
T.Chol (mg/dL)	38.20 ± 7.92	39.20 ± 10.26	37.80 ± 7.29	41.80 ± 4.97	35.00 ± 6.40	48.00 ± 7.04#
Trig (mg/dL)	79.60 ± 66.59	91.80 ± 63.38	58.20 ± 13.14	65.80 ± 22.59	77.20 ± 32.48	82.60 ± 34.96
T.Bil (mg/dL)	0.06 ± 0.03	0.05 ± 0.03	0.05 ± 0.04	0.09 ± 0.03	0.11 ± 0.02	0.10 ± 0.05
AST (U/L)^$^	80.00 ± 4.95	96.60 ± 6.47^*∗*^	90.20 ± 7.19^*∗*^	81.20 ± 5.72	89.00 ± 9.35	95.80 ± 25.79
ALT (U/L)^$^	31.40 ± 1.95	35.40 ± 5.18	39.4 ± 4.93	46.00 ± 11.11^*∗*^	38.80 ± 6.83	35.20 ± 8.26
ALP (U/L)	39.80 ± 8.76	49.00 ± 15.73	41.00 ± 5.70	47.40 ± 13.74	37.40 ± 16.32	36.20 ± 14.31
TP (g/dL)	6.79 ± 0.22	6.82 ± 0.40	6.66 ± 0.38	6.80 ± 0.29	6.98 ± 0.19	7.18 ± 0.27
Alb (g/dL)	4.38 ± 0.15	4.28 ± 0.19	4.28 ± 0.27	4.34 ± 0.26	4.30 ± 0.14	4.24 ± 0.28
Ca (mg/dL)	10.56 ± 0.25	10.10 ± 0.19	10.20 ± 0.21	10.38 ± 0.49	10.48 ± 0.13	10.44 ± 0.17
Phos (mg/dL)	4.88 ± 0.50	4.46 ± 0.23	5.30 ± 0.52	5.16 ± 0.29	4.16 ± 0.48	4.68 ± 0.66
Na (mmoL/L)	141.16 ± 1.03	141.74 ± 0.65	140.44 ± 0.96	141.46 ± 1.02	141.70 ± 0.69	141.74 ± 0.65
K (mmoL/L)	3.50 ± 0.28	3.37 ± 0.15	3.65 ± 0.19	3.54 ± 0.15	3.52 ± 0.20	3.59 ± 0.17
Cl (mmoL/L)	105.54 ± 1.33	105.82 ± 0.79	104.04 ± 0.91	105.54 ± 1.16	106.24 ± 0.91	105.54 ± 1.77

Data are present as mean ± SD; *n* = 5. ^*∗*^ and #Significance *p* < 0.05 vs. G1 and G1R, respectively. ^$^The mean ± SD of the historical values of AST is 107.75 ± 33.30 U/L (min. 70.0 U/L; max. 167.0 U/L) and ALT is 41.47 ± 9.08 U/L (min. 25.0 U/L; 67.0 U/L).

**Table 11:** Summary of histopathological observations on the vital organs of male Wistar rats after 28 days of oral supplementation of GMCT.

Organs/findings	Number of animals showing abnormal findings (severity score)	
	GMCT dose (mg/kg/day)	
	0 (G1)	1000 (G4)
Adrenals		
Cortical tissue, accessory	1 (+)	1 (+)
Cecum		
Infiltration, MNC, mucosa	1 (+)	1 (+)
Kidneys		
Mineralization, tubular, outer medulla	0	1 (+)
Liver	0	0
Lungs		
Haemorrhage, alveolar	0	1 (++)
Mineralization/bone formation, alveolar	1 (+)	0
Pancreas		
Degeneration/atrophy, acinar	1 (+)	1 (+)
Pituitary gland		
Distension/secretion, Rathke's cleft	1 (+)	0
Pseudocyst, pars intermedia	1 (+)	0
Thymus		
Haemorrhage, medulla	0	1 (+)
Haemorrhage, medulla	1 (++)	0
Necrosis, lymphocytes, cortex	0	1 (++)
Trachea		
Glandular dilation, submucosa	1 (+)	0
Urinary bladder		
Eosinophilic material (seminal plug), lumen	1 (++)	0
Luminal dilation	0	1 (++)
Testes		
Mineralization and degeneration, seminiferous epithelium, tubular	0	1 (+)
Epididymis		
Infiltration, MNC, interstitial	0	1 (+)
Prostate gland		
Infiltration, MNC, interstitial	0	1 (+)

*n* = 5; scores (+) and (++) indicate minimal and mild abnormality, respectively.

**Table 12:** Summary of histopathological observations on the vital organs of female Wistar rats after 28 days of oral supplementation of GMCT.

Organs/findings	Number of animals showing abnormal findings (severity score)	
	GMCT dose (mg/kg/day)	
	0 (G1)	1000 (G4)
Adrenals		
Cortical tissue, accessory	1 (+)	2 (+)
Kidneys		
Mineralization, tubular, outer medulla	0	1 (+)
Liver	0	0
Lungs		
Mineralization	1 (+)	0
Alveolar infiltration, MNC, perivascular	1 (+)	0
Pituitary gland		
Pseudocyst, pars intermedia	0	1 (+)
Thymus	0	0
Haemorrhage, medulla	1 (+)	0
Necrosis, lymphocytes, cortex	1 (++)	0
Thyroid		
Thymic tissue, ectopic	1 (+)	0
Trachea		
Glandular dilation, submucosal	0	1 (+)
Uterus		
Luminal dilatation (proestrous stage)	1 (+)	1 (+)

*n* = 5; scores (+) and (++) indicate minimal and mild abnormality, respectively.

**Table 13:** *In vitro* mutagenicity assay of GMCT in the Ames test.

Treatment (*μ*g/plate)	Number of revertant colonies/plate (mean ± SD)									
	TA98 (+S9)	TA98 (−S9)	TA100 (+S9)	TA100 (−S9)	TA1535 (+S9)	TA1535 (−S9)	TA1537 (+S9)	TA1537 (−S9)	WP2 uvrA/(pKM101) (+S9)	WP2 uvrA/pKM101 (−S9)
Vehicle control (0)	25.7 ± 3.1	22.3 ± 2.5	124.0 ± 3.6	123.3 ± 3.5	13.7 ± 1.5	11 ± 2	9.3 ± 2.5	6.3 ± 1.5	132.3 ± 4.9	130.3 ± 3
100	21.3 ± 3.5	22.3 ± 1.5	122.3 ± 2.5	125.3 ± 3.5	10.3 ± 1.5	11.7 ± 1.5	6.3 ± 1.5	7 ± 2	123 ± 3	124 ± 3.6
266	25.3 ± 2.5	22.7 ± 2.5	123.7 ± 3.1	124.3 ± 4	9.3 ± 2.1	8 ± 2	6.7 ± 1.5	6.3 ± 1.5	124 ± 2.6	123 ± 3.6
707	22.7 ± 2.1	21 ± 2	124.7 ± 2.5	125 ± 2	8 ± 1	8.3 ± 1.5	8 ± 2	6 ± 1	123 ± 1	124.3 ± 1.5
1880	22 ± 3.6	21 ± 3.6	123 ± 2	123.3 ± 2.5	8.7 ± 1.5	10 ± 1	7 ± 1	5.3 ± 1.5	121 ± 1	122.3 ± 2.1
5000	22.7 ± 3.5	20.7 ± 1.5	123 ± 4.4	124 ± 3.6	8.7 ± 2.1	9.3 ± 2.1	9 ± 2	5.7 ± 2.1	120.3 ± 1.5	123.3 ± 3.2
Positive control	549 ± 17.7^a^	289.7 ± 13^c^	880.3 ± 18.5^a^	565.3 ± 14^d^	142 ± 8.9^a^	152.7 ± 9.3^d^	142.3 ± 7.5^a^	144 ± 6.6^e^	575.7 ± 11.7^b^	542.7 ± 9.6^f^

^a^2-Aminoanthracene (4 *μ*g/plate); ^b^2-aminoanthracene (30 *μ*g/plate); ^c^2-nitrofluorence (2 *μ*g/plate); ^d^sodium azide (1 *μ*g/plate); ^e^9-aminoacridine (50 *μ*g/plate); ^f^4-nitroquinoline-1-oxide (4 *μ*g/plate).

**Table 14:** Effect of GMCT supplementation on micronucleus frequency in mouse bone marrow erythrocytes.

Treatments	Dose (mg/kg)	Male (*n* = 5)		Female (*n* = 5)	
		PCE : TE	% MNPCE	PCE : TE	% MNPCE
Vehicle control	0	0.642 ± 0.038	0.080 ± 0.048	0.591 ± 0.021	0.110 ± 0.058
GMCT	500	0.625 ± 0.036	0.0035 ± 0.034	0.595 ± 0.041	0.025 ± 0.035
GMCT	1,000	0.612 ± 0.030	0.020 ± 0.033	0.616 ± 0.025	0.040 ± 0.038
GMCT	2,000	0.600 ± 0.032	0.015 ± 0.022	0.616 ± 0.029	0.020 ± 0.027
Cyclophosphamide monohydrate	40	0.618 ± 0.045	1.900 ± 0.083^*∗*^	0.612 ± 0.038	1.910 ± 0.068^*∗*^

Data are present as mean ± SD; ^*∗*^*p* < 0.05, comparison between treatment and control groups in one-way ANOVA. MNPCE = micronucleated polychromatic erythrocyte; PCE = polychromatic erythrocytes; TE = total erythrocytes.

## Data Availability

The data used to support the findings of this study are available upon request.

## Abbreviations

ALT: Alanine aminotransferase
AST: Aspartate aminotransferase
BW: Body weight
CPCSEA: Committee for the purpose of control and supervision of experiments on animals
HPLC: High-performance liquid chromatography
LD50: Median lethal dose
MNPCE: Micronucleated polychromatic erythrocytes
NOAEL: No-observed-adverse-effect level
OECD: Organization of Economic Cooperation and Development
PCE: Polychromatic erythrocytes.
